# Synthesis and Anticancer Activity of Bagasse Xylan/Resveratrol Graft-Esterified Composite Nanoderivative

**DOI:** 10.3390/ma15155166

**Published:** 2022-07-26

**Authors:** Bin Zhao, Heping Li, Yue Su, Kexin Tian, Zhiming Zou, Wenli Wang

**Affiliations:** 1 College of Chemistry and Bioengineering, Guilin University of Technology, Guilin 541004, China; z1175325759@163.com (B.Z.); sy522853325@163.com (Y.S.); tian1040034842@163.com (K.T.); 357921449@163.com (Z.Z.); 2 College of Textile and Clothing Engineering, China National Textile and Apparel Council Key Laboratory of Natural Dyes, Soochow University, Suzhou 215123, China

**Keywords:** bagasse xylan/resveratrol nanoderivative, synthesis, graft esterification, biological docking, anticancer activity

## Abstract

Biomass materials are high-quality raw materials for the preparation of natural, green and highly active functional materials due to their rich active groups, wide sources and low toxicity. Bagasse xylan (BX) and resveratrol (Res) were used as raw materials to introduce ethylene glycol dimethacrylate (EGDMA) via grafting reaction to obtain the intermediate product BX/Res-g-EGDMA. The intermediate was esterified with 3-carboxyphenylboronic acid (3-CBA) to obtain the target product 3-CBA-BX/Res-g-EGDMA. The BX/Res-composite-modified nanoderivative with antitumor activity was synthesized with the nanoprecipitation method. The effects of the reaction conditions on the grafting rate (*G*) of BX/Res-g-EGDMA and the degree of substitution (*DS*) of 3-CBA-BX/Res-g-EGDMA were investigated using single-factor experiments. The results showed that under the optimized process conditions, *G* and *DS* reached 142.44% and 0.485, respectively. The product was characterized with FTIR, XRD, TG-FTC, ^1^H NMR and SEM, and its anticancer activity was simulated and tested. The results showed that 3-CBA-BX/Res-g-EGDMA had a spherical structure with an average particle size of about 100 nm and that its crystalline structure and thermal stability were different from those of the raw materials. In addition, 3-CBA-BX/Res-g-EGDMA showed the best docking activity with 2HE7 with a binding free energy of −6.3 kJ/mol. The inhibition rate of 3-CBA-BX/Res-g-EGDMA on MGC80-3 (gastric cancer cells) reached 36.71 ± 4.93%, which was 18 times higher than that of BX. Therefore, this material could be a potential candidate for biomedical applications.

## 1. Introduction

In recent years, natural biomass has attracted much attention from researchers because of its structural diversity and self-contained bioactivity [1,2]. The development of and research on compounds with potential biological activity through chemical modifications [3] such as grafting [4], cross-linking [5] and esterification [6] using biomass resources as lead compounds have enormous research significance and potential application prospects.

Cancer is the second leading cause of death worldwide after cardiovascular disease. Chemotherapy is currently the standard treatment for cancer. Although conventional chemotherapeutic agents may be cytotoxic to rapidly proliferating cancer cells, serious adverse effects are commonly encountered. Due to these side effects and the potential development of cancer-cell resistance, it is important to develop novel, non-toxic biomass materials [7]. Specifically, polysaccharides and polyphenols are of interest due to their potential in the prevention of various diseases [8]. Bagasse xylan (BX), as an important active substance of natural, green and renewable resources, has a large number of active hydroxyl groups at the C2 and C3 positions of the main chain xylose repeating units, as well as its side-chain structure containing multiple active groups [9,10,11]. Resveratrol (Res), extracted from natural plants such as peanut and grape, is a very biologically active polyphenolic compound with the C5,5 phenolic hydroxyl group as the active center, which can be modified via esterification and etherification [12,13]. These special groups give bagasse xylan, resveratrol and their derivatives good biological activity. The unique structure and strong bioactivity of bagasse xylan and resveratrol have shown remarkable effects in antitumor [14,15], antivirus [16] and antioxidant applications [17]. At present, research on biomass materials mostly adopts a single modification method to improve their functionality [18,19,20], but the modified substances still suffer from low bioactivity, poor biocompatibility and low utilization. The use of composite modifications may be the means to make up for some of these defects [21]. Qian et al. [22] synthesized xylan derivatives with various chemical modifications and performed molecular-docking and activity tests. The results showed that the synthesized derivatives inhibited a wide range of tumor cells and that multiple group modifications were more effective in enhancing the antitumor activity of xylans. Therefore, it is very important to adopt composite modification means that can help to maximize the yield while minimizing the BX and Res limitations.

In recent years, results were achieved at home and abroad in exploring synergistic antioxidant, antitumor and enhanced biological activities [23,24,25]. Ozdemir et al. [26] Explored the apoptotic effects of cisplatin (CDDP) and Res alone or in combination for the treatment of MDA-MB-231 cells and determined the toxic effects of the drugs on MDA-MB-231 cells with MTT assays, which showed that the combination had stronger antitumor activity. In a previous study, Res was shown to act synergistically with dihydroartemisinin (DHA) in HepG2 and MDA-MB-231 cancer cells, and the combination controlled the migration of cancer cells with a stronger inhibitory effect than with Res and DHA alone [27]. This suggests that when multiple substances with antitumor activity are used in combination, the effect may be even stronger [28,29,30], which suggests the potential value of studying the synergistic effects of various compounds and their composite modification.

In this paper, based on the existing studies on the antitumor activity of BX and Res, BX and Res were mixed to a certain ratio. This was also modified via grafting, cross-linking and esterification to synergistically improve the antitumor and antiviral activities using the effective superposition of chemical components. The active macromolecules were prepared into nanoparticles with the nanoprecipitation method to obtain novel anticancer active nanoderivatives to broaden their application and provide a reference for green, natural and safe functional materials, drug carriers and drug precursors with antitumor activity.

## 2. Materials and Methods

### 2.1. Materials

BX was isolated from bagasse (self-extracted). Emulsifier OP-10, hydrochloric acid and sodium hydroxide were acquired from Xilong Chemical Factory (Shantou, China). Resveratrol was obtained from Genye Biotechnology Company limited (Shanghai, China). 3-Carboxyphenylboronic acid, vinyl dimethacrylate, 4-dimethylaminopyridine, 1-(3-dimethylaminopropyl)-3-ethylcarbodiimide hydrochloride, ammonium persulfate (APS), N, N-dimethylformamide (DMF) and N, N-methylene acrylamide were obtained from Macklin (Shanghai, China). Ethanol and dimethyl sulfoxide (DMSO) were purchased from Luoyang Chemical Reagent Factory (Luoyang, China).

### 2.2. Preparation of Initiator Solution

APS (0.6 g) was placed in a beaker with 20 mL of distilled water and shaken ultrasonically for 5–8 min until the ammonium persulfate was fully dissolved to form an initiator solution, and the initiator solution was subsequently poured into a 100 mL constant-pressure dropping funnel.

### 2.3. Preparation of EGDMA Monomer Emulsion

At first, EGDMA (0.4 mL) was mixed in a beaker containing 30 mL of distilled water; then, OP-10 (1.5 mL) was added and stirred for 5 min to form a homogeneous emulsion, which was then poured into a 100 mL constant-pressure dropping funnel.

### 2.4. Synthesis of BX/Res–EGDMA Grafted Copolymer

BX/Res was modified with EGDMA using graft copolymerization. First, BX (2.4 g) and Res (0.6 g) were added to a four-necked flask equipped with a thermometer, a condensing reflux device and 50 mL of distilled water, and the temperature was raised to 50 °C; the liquid was stirred for 0.5 h. Then, the temperature was raised to 65 °C, and the APS solution was added dropwise; after a period of dropwise addition, the monomer emulsion and the initiator solution were added dropwise at the same time for a controlled time of 4.0 h. After the reaction was completed, the mixture of BX/Res–EGDMA copolymer was cooled to room temperature, filtered and finally washed with ethanol four times for purification. The obtained BX/Res–EGDMA graft copolymer was dried in an oven at 60 °C for 24 h.

**Figure 1:**
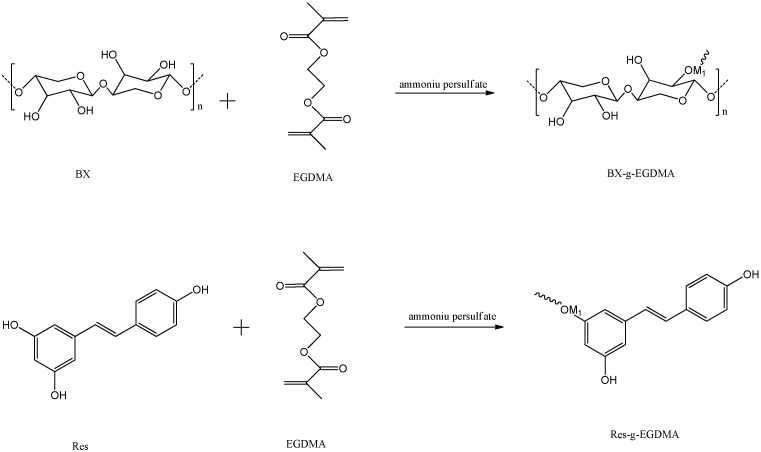

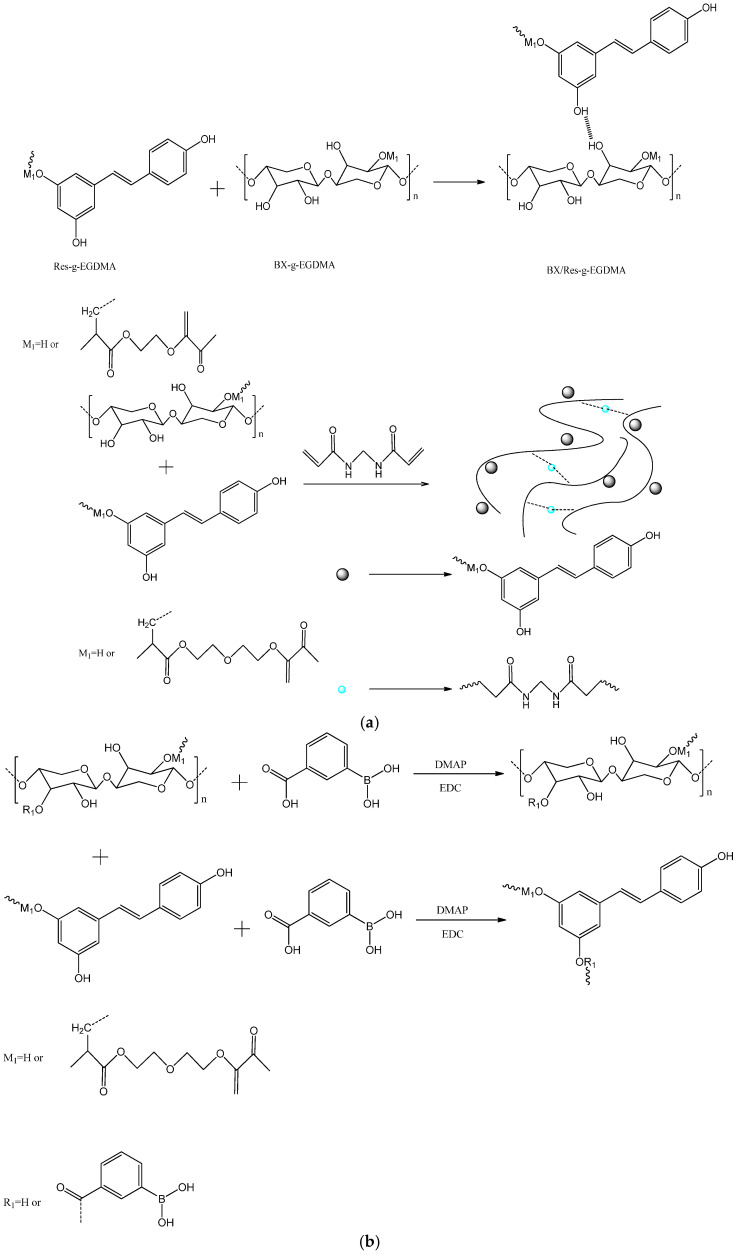
(**a**) The synthesis routes of BX/Res-g-EGDMA and (**b**) 3-CBA-BX/Res-g-EGDMA.

A schema of the chemical modification of BX/Res with EGDMA is represented in Figure 1a.

### 2.5. Synthesis of Esterified Derivatives of 3-CBA-BX/Res-g-EGDMA

BX/Res-g-EGDMA was modified via esterification with 3-CBA. First, BX/Res-g-EGDMA (1.5 g) and DMSO (60 mL) were added to a four-necked flask equipped with a magnetic stirrer and thermometer, heated to 60 °C and stirred until complete dissolution; then, 3-CBA (2.5 g), 4-dimethylaminopyridine (0.2 g) and EDC (0.2 g) were added sequentially and stirred for 6.0 h to complete the reaction, and the system was cooled to room temperature [31,32].

Subsequently, acetone (80 mL) was added to the cooled system for precipitation, and this was allowed to stand for 30 min until the upper and lower layers were separated; then, it was filtered. The obtained filter cake was washed three times with 50 mL of ethanol and 70 mL of distilled water, respectively, and then sent to a vacuum thermostatic oven at 60 °C for 24 h to obtain 3-CBA-BX/Res-g-EGDMA.

A schema of the functionalization of BX/Res–EGDMA with 3-CBA is represented in Figure 1b.

### 2.6. Preparation of Nanoparticles 3-CBA-BX/Res-g-EGDMA

Nanoparticles were prepared with the nanoprecipitation method. In short, we placed 3-CBA-BX/Res-g-EGDMA (0.6 g) in a single flask containing 50 mL of dimethyl sulfoxide. The temperature was raised to 70 °C and stirred until complete dissolution. The system was then cooled to room temperature, and the liquid was slowly dripped into rapidly stirring absolute ethanol; the resulting suspension was transferred to a centrifuge jar for centrifugation. After centrifugation, the absolute ethanol was filtered out, and centrifugation was continued with the addition of distilled water. After centrifugation, 3-CBA-BX/Res-g-EGDMA was removed, freeze-dried to constant weight in a vacuum freeze-dryer, removed and ground to obtain 3-CBA-BX/Res-g-EGDMA nanoparticles.

### 2.7. Determination of Grafting Rate and Monomer Conversion Rate

Impurities were removed from BX/Res-g-EGDMA via soxhlet extraction with cyclohexane for 24 h.

The grafting rate (*G*) and grafting efficiency (*GE*) of BX/Res-g-EGDMA were calculated as follows [33]:

(1) $$\;G=\frac{W_{2}}{W_{0}}\times 100\%$$

(2) $$GE=\frac{W_{2}}{W_{1}}\times 100\%$$

where *W*_0_ is the weight of BX, *W*_1_ is the weight of the monomer and *W*_2_ is the weight of the grafted branch chain.

### 2.8. Determination of Degree of Substitution

The acid–base titration method is a classical method for determining the degree of esterification substitution of polymers [34], and the specific experimental procedure for this method is as follows: Weigh 0.20 g of 3-CBA-BX/Res-g-EGDMA in a 25 mL conical flask; then, add 5 mL of distilled water to the conical flask. Mix well; then, add 2 drops of phenolphthalein reagent and shake well. Drop with 0.10 mol/L NaOH standard solution until slightly red (the color does not fade within half a minute). Then, add 2.5 mL of NaOH standard solution with a concentration of 0.5 mol/L, shake well and saponify with electromagnetic stirring at room temperature for 4.0 h; finally, titrate with HCl standard solution with a concentration of 0.50 mol/L until it just turns colorless [35]:

(3) $$w_{c}=\frac{\left(V_{0}-V_{1}\right)\times 10^{-3}\times C_{HCl}\times M}{m}$$

(4) $$\;DS=\frac{182\times w_{c}}{M-\left(M-1\right)\times w_{c}}\;\;\;$$

where *V*_0_ (mL) is the volume of HCl standard solution (0.5 mol/L) used in the blank experiment; *V*_1_ (mL) is the volume of HCl standard solution (0.5 mol/L) used for the titration of 3-CBA-BX/Res-g-EGDMA; *C*_HCl_ is the concentration of HCl standard solution; *m* (g) is the mass of the target product sample; *M* (g/mol) is the molar mass of carboxylic acid acyl; 182 (g/mol) is the relative molecular mass of the dehydration unit of BX/Res; *w*_c_ (%) is the mass fraction of carboxylic acid acyl in 3-CBA-BX/Res-g-EGDMA.

### 2.9. Characterization

The product was mixed with KBr at a ratio of 1:50–100 (*w*/*w*), ground and mixed well, and the FT-IR spectra of the samples were recorded using a Nicolet-iSL0 Fourier-transform infrared spectrometer from TA Instruments Co., Ltd. (New Castle, DE, USA). The morphology of the samples was imaged using a Quanta 450 scanning electron microscope. The XRD curves of the samples were obtained using X’Pert PRO (PANalytical B.V., Amelo, The Netherlands) at 40 kV and 55 mA using Cu Kα radiation manipulation. The samples were analyzed for thermal degradation using an SDT-Q600 simultaneous TGA/DSC analyzer from TA Instruments Co., Ltd. (New Castle, DE, USA) at a temperature range of 25–800 °C with a ramp rate of 10 °C/min. TG (thermogravimetric analysis) and DTG (differential thermogravimetric analysis) of the samples were conducted. The samples were tested with ^1^H NMR using an AVANCE 500 MHz NMR hydrogen spectrometer from Bruker, Romanshorn, Switzerland. The samples to be tested were dissolved in deuterated DMSO solvent, and the samples of BX, Res and target products were tested at 500 MHz and 298.2 K.

### 2.10. Molecular-Docking Study

Molecular docking was used to evaluate the binding ability of ligand molecules and receptor proteins based on the principles of energy matching, geometry matching and chemical-environment matching and to predict the best binding mode between them, the optimal confirmation, so that the free energy of the whole system was minimized. A molecular-docking study was performed using a module integrated into Autodock molecular modeling software. The structures of 2HE7, 5JQI, 5ZSY and 6YAU were obtained from Protein Data Bank (http://www.rcsb.org/pdb, accessed on 26 March 2022) [36,37].

### 2.11. Tumor-Cell-Proliferation Inhibitory Assay

The effects of 3-CBA-BX/Res-g-EGDMA on the proliferation profile of MGC80-3 (human gastric cancer cells), BEL-7407 (human liver cancer cells), NCI-H460 (human lung cancer cells) and NGEC (normal gastric mucosa epithelial cells) were investigated using the MTT method. The optical density (*OD*) was measured at 490 nm as a test, and the *OD* was measured at 630 nm as a reference. Blank experiments included only culture medium, MTT and DMSO. Control experiments included cells, culture medium, MTT and DMSO. Sample experiments included material, cells, culture medium, MTT and DMSO. All experiments and measurements were performed in triplicate, and approximate averages were taken throughout the data analysis and calculations. The results were statistically analyzed using Microsoft Office Excel 2010 and statistical software packages. The inhibition rate of these compounds on cancer cells was calculated using the following equation [38]:

(5) $$\begin{array}{c} Relative\;Cell\;Proliferation\;Ratio\left(RCR\%\right)\\ \;=\frac{OD_{\left(\mathrm{sample},490\mathrm{nm}-630\mathrm{nm}\right)}-OD_{\left(\mathrm{blank},490\mathrm{nm}-630\mathrm{nm}\right)}}{OD_{\left(\mathrm{control},490\mathrm{nm}-630\mathrm{nm}\right)}-OD_{\left(\mathrm{blank},490\mathrm{nm}-630\mathrm{nm}\right)}}\times 100\%\; \end{array}$$

inhibition ratio = 1 − *RCR* %.

In tests, the standard deviation (*SD*) was estimated, and experiments were repeated to determine the experimental error. All data were reported as means ± standard deviation.

## 3. Results and Discussion

### 3.1. Single-Factor Analysis of Graft Copolymerization Reaction

**Figure 2:**
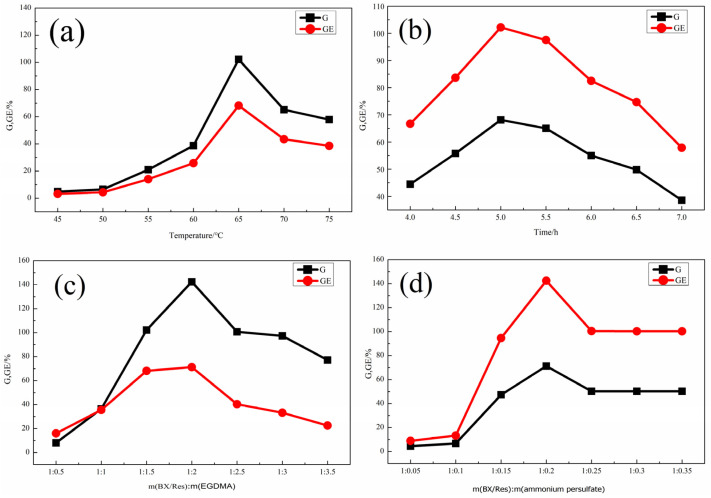
(**a**) The effect of reaction temperature on *G* and *GE*. (**b**) The effect of reaction time on *G* and *GE.* (**c**) The effect of m(BX/Res):m(EGDMA) on *G* and *GE*. (**d**) The effect of the mass of ammonium persulfate on *G* and *GE*.

The effects of the reaction temperature, reaction time, mass ratio of ammonium persulfate to BX/Res and mass ratio of EGDMA to BX/Res on graft copolymerization were investigated. As shown in Figure 2, the effects of the above factors were evaluated while keeping the other three factors constant. The results showed that the optimal reaction temperature was 65 °C and that the optimal grafting time was 5.0 h. The optimal mass ratio m(BX):m(potassium persulfate) obtained from the curve in Figure 2c was 1:2, and the optimal mass ratio m(BX/Res):m(ammonium persulfate) obtained from the curve in Figure 2d was 1:0.2. Under optimal reaction conditions, the *G* and *GE* of BX/Res-g-EGDMA reached, respectively, 142.44% and 71.22%.

### 3.2. Single-Factor Analysis of Esterification Reaction

**Figure 3:**
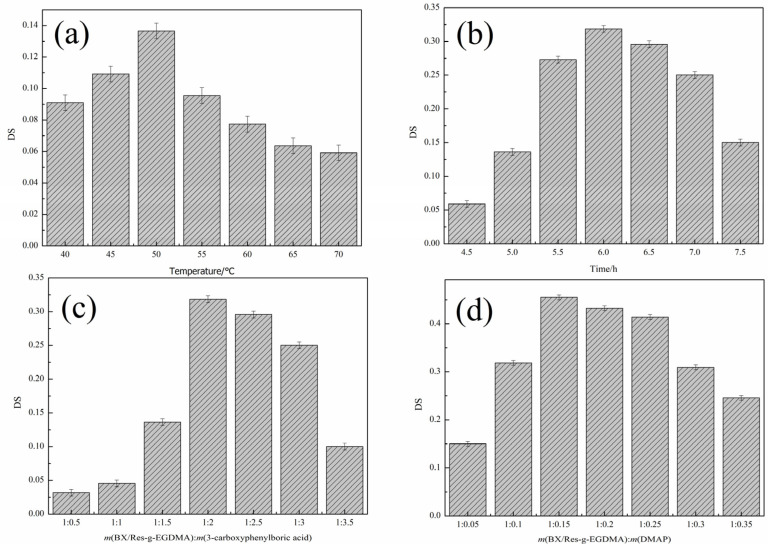
Effects of reaction conditions on *DS*. (**a**) The effect of reaction temperature on *DS*. (**b**) The effect of reaction time on *DS*. (**c**) The effect of m(BX/Res-g-EGDMA):m(3-carboxyphenylboric acid) on *DS*. (**d**) The effect of m(BX/Res-g-EGDMA):m(DMAP) on *DS*.

In order to improve the degree of esterification, four variables were considered: esterification temperature, esterification time, the mass ratio of BX/Res-g-EGDMA to 3-carboxyphenylboron and the mass ratio of BX /Res-g-EGDMA to DMAP. As shown in Figure 3, the influence of the above factors was evaluated while keeping the other three factors unchanged. The conclusions were that the optimum reaction temperature was 50 °C and that the optimum reaction time was 6.0 h. As shown in Figure 3c,d, the best mass ratio of BX/Res-g-EGDMA to 3-carboxyphenylboric acid was 1:2, and for m(BX/Res-g-EGDMA):m(DMAP), this was 1:0.15, which was the best feed ratio. Under the best reaction conditions, *DS* reached 0.485.

### 3.3. Structure Analyses

Based on the single-factor experiments, the raw materials and target products were subsequently characterized using FTIR, TG–DTG, XRD, SEM, ^1^H NMR and other performance tests.

#### 3.3.1. FTIR Analyses

**Figure 4:**
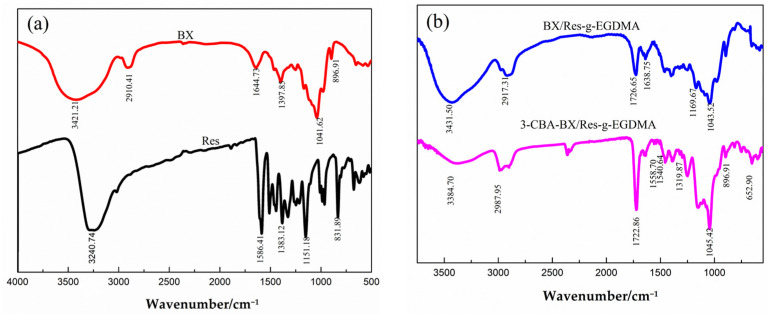
Spectra of BX, Res, BX/Res-g-EGDMA and 3-CBA-BX/Res-g-EGDMA. (**a**) FTIR spectra of BX and Res. (**b**) TIR spectra of BX/Res-g-EGDMA and 3-CBA-BX/Res-g-EGDMA.

The functional groups in the synthesized samples were identified using FTIR spectroscopy; the spectra are shown in Figure 4. In the spectrum of bagasse xylan, the broad peaks at 3421.21 cm^−1^ and 2910.41 cm^−1^ corresponded to —OH and C—H stretching vibrations, respectively. The absorption peak of the BX molecular backbone vibration was 896.91 cm^−1^, while the peaks of Res were basically those of the benzene ring and hydroxyl group as well as the C=C double bond. After grafting and cross-linking, a new peak appeared in the BX/Res-g-EGDNA spectrum at 1726.65 cm^−1^ and was attributed to the C=O stretching vibration of the ester carbonyl group in EGDMA. The peak at 638.75 cm^−1^ was assigned to the N—H stretching vibration, and the peaks centered at 1043.52 cm^−1^ and 1169.67 cm^−1^ were attributed to the stretching vibration of acrylate C—O. The results showed that this BX/Res graft copolymer possessed the characteristic peak of EGDMA. As shown by the IR spectrum of 3-CBA-BX/Res-g-EGDMA, compared with the BX/Res-g-EGDMA spectrum, new benzene-ring-backbone vibrational peaks appeared at 1558.70 cm^−1^ and 1540.64 cm^−1^, and the peak at 1319.87 cm^−1^ corresponded to the B—O bending vibrations. These peaks confirmed that the target product contained the characteristic IR absorption peaks of EGDMA and 3-CBA.

#### 3.3.2. XRD Analyses

**Figure 5:**
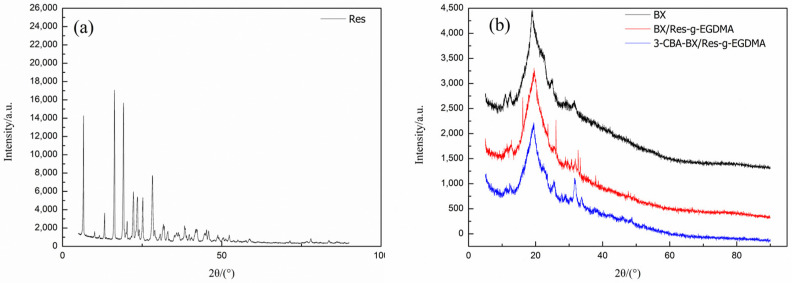
(**a**) XRD patterns of Res. (**b**) XRD patterns of BX, BX/Res-g-EGDMA and 3-CBA-BX/Res-g-EGDMA.

XRD was used to identify the crystalline phases of BX, Res, BX/Res-g-EGDMA and 3-CBA-BX/Res-g-EGDMA; the XRD patterns are shown in Figure 5. The XRD pattern of the BX sample showed that the diffraction peaks of BX appeared at 12.62°, 14.96°, 19.16°, 23.86° and 31.67°; especially, at 19.16°, a broader diffraction peak was observed, while the other peak patterns showed to be relatively weak, indicating the low crystallinity of bagasse xylan itself. Moreover, as shown in Figure 5a, the XRD pattern of Res showed peaks at 2θ angles of 6.73°, 16.45°, 19.12°, 25.3°, 28.49°, 31.65° and 33.02°, which indicated that Res had a high crystallinity. The XRD pattern showed that the main characteristic peak of BX/Res-g-EGDMA (19.16°) was consistent with BX, while sharp diffraction peaks appeared at 16.17°, 25.34°, 28.78°, 31.64° and 33.74° compared with the BX sample. The positions of the diffraction peaks were the same as several characteristic peaks appearing in Res, indicating the presence of BX/Res-g-EGDMA produced by grafting and cross-linking reactions between the semicrystalline-term material BX and the highly crystalline-phase material Res. BX/Res-g-EGDMA tended to be similar to the semicrystalline material. Compared with BX/Res-g-EGDMA, the diffraction peaks of 3-CBA-BX/Res-g-EGDMA had the same positions, and the main characteristic peak (19.16°) of 3-CBA-BX/Res-g-EGDMA was consistent with BX/Res-g-EGDMA. However, the peak shape of product 3-CBA-BX/Res–EGDMA changed after the esterification reaction of BX/Res-g-EGDMA, and the overall diffraction peak became broader and flatter. The crystallinity became lower. 3-CBA-BX/Res-g-EGDMA tended to be similar to the semicrystalline material.

#### 3.3.3. SEM Analyses of 3-CBA-BX/Res-g-EGDMA

**Figure 6:**
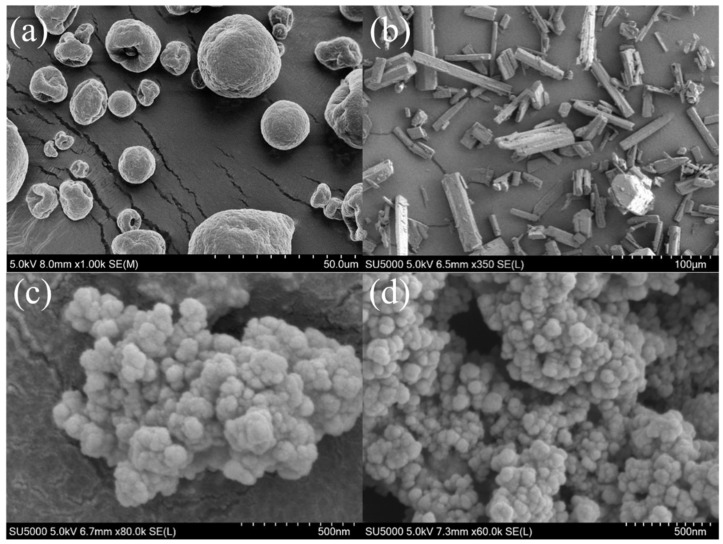
SEM images of BX, Res, BX/Res-g-EGDMA and 3-CBA-BX/Res-g-EGDMA. (**a**) SEM image of BX. (**a**) SEM image of BX. (**b**) SEM image of Res. (**c**) SEM image of BX/Res-g-EGDMA. (**d**) SEM image of 3-CBA-BX/Res-g-EGDMA.

The surface characteristics of the samples were investigated using scanning electron microscopy (SEM). The SEM images in Figure 6 show the morphology of BX, Res, BX/Res-g-EGDMA and 3-CBA-BX/Res-g-EGDMA. As shown in Figure 6a, the morphology of BX was a smooth spherical particle with a mostly unchanged spherical shape and a diameter of about 10–20 μm. Figure 6b shows the morphological characteristics of Res with a smooth, rod-shaped surface and a diameter of about 5–10 μm. As can be seen in Figure 6c, the morphology of BX/Res-g-EGDMA changed to an irregular spherical shape with a non-uniform arrangement of cloud-like particles. As shown in Figure 6d, after the esterification of BX/Res-g-EGDMA with 3-CBA, the cloud-like particles broke up into smaller and more dense particles and most of the aggregate into fish-egg-like structures with an average particle size of about 50–100 nm.

#### 3.3.4. TG–DTG Analyses of 3-CBA-BX/Res-g-EGDMA

**Figure 7:**
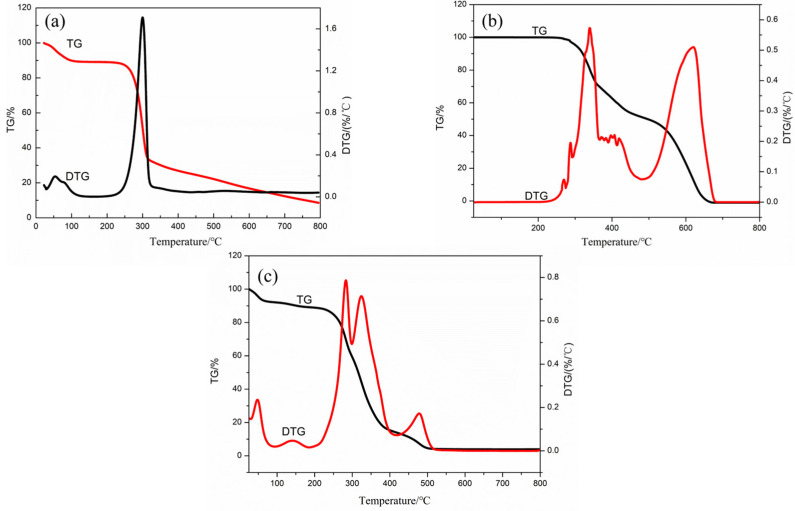
TG–DTG curves of BX, Res, and 3-CBA-BX/Res-g-EGDMA. (**a**) TG–DTG curve of BX. (**b**) TG–DTG curve of Res. (**c**) TG–DTG curve of 3-CBA-BX/Res-g-EGDMA.

The TG–DTG curves of BX, Res and 3-CBA-BX/Res-g-EGDMA are shown in Figure 7. From the TG–DTG curves of Res, it could be seen that the process of mass change due to the increase in temperature in the test range of 0–800 °C could be divided into two stages. In the first stage, in the range of 280–500 °C, the mass loss was about 60%, probably due to the breakage of hydroxyl and unsaturated double bonds of Res leading to the decomposition of Res. In the second stage, the temperature reached higher values, and Res was gradually completely decomposed, probably due to the disruption of the ring structure of resveratrol.

In Figure 7b, the front end of the TG–DTG curve of BX showed a small amount of mass loss (Figure 7b; about 12.16% mass loss). These losses were related to the evaporation of water molecules physically adsorbed on the surface of the sample; the amount of mass loss in the range of 250–325 °C was approximately 60% and was probably due to the breakage of crystalline water and hydroxyl groups on xylan. The loss of bagasse xylan was about 17% in the range of 325–800 °C, probably due to branched-chain residues, the hydroxyl groups on bagasse xylan, the breakage of the main chain of bagasse xylan, etc.

It can be seen from Figure 7c that the process of 3-CBA-BX/Res-g-EGDMA mass changing due to the temperature rise could be divided into five stages within the test range of 25–800 °C. Firstly, in the range of 25–100 °C, the mass loss of 3-CBA-BX/Res-g-EGDMA was about 10%, which was caused by the water loss in the sample. In the second stage, in the range of 100–250 °C, the mass loss was about 2%, which may have been caused by the loss of crystal water and unreacted hydroxyl fracture. In the third stage, the mass loss was about 56% at 250–375 °C, which may have been due to the breaking of some bonds, such as some residues and hydroxyl groups on xylan and the breaking of the xylan main chain. In the fourth stage of 375–500 °C, the mass loss of 20% may have been caused by the fracture of the double bond, crosslinker or ester bond in EGDMA. The fifth stage was 500–800 °C, and the quality remained unchanged. The TG–DTG analyses showed that the thermal stability of the modified final product changed compared with the raw materials.

#### 3.3.5. ^1^H NMR Analyses of 3-CBA-BX/Res-g-EGDMA

**Figure 8:**
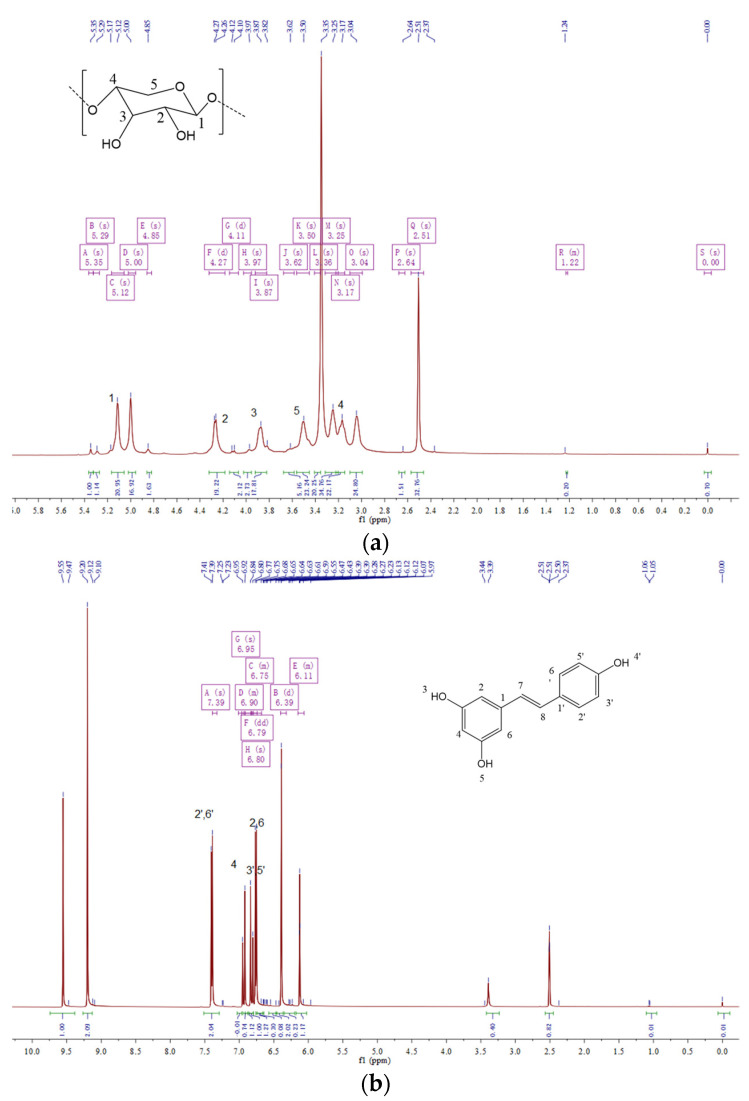

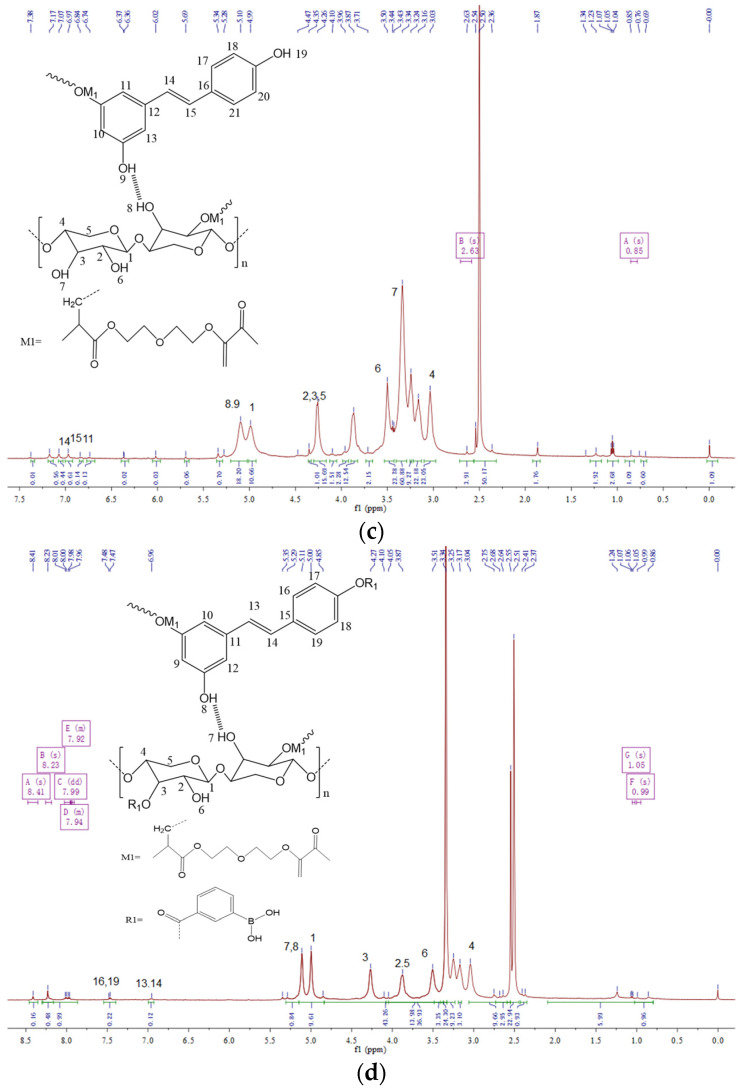
(**a**) ^1^H NMR spectrum of BX. (**b**) ^1^H NMR spectrum of Res. (**c**) ^1^H NMR spectrum of BX/Res-g-EGDMA. (**d**) ^1^H NMR spectrum of 3-CBA-BX/Res-g-EGDMA.

The structures of BX, Res and the synthesized BX/Res-g-EGDMA and 3-CBA-BX/Res-g-EGDMA were characterized using ^1^H NMR as shown in Figure 8. In all ^1^H NMR maps, the proton peaks with chemical shift δ = 0 were TMS peaks, and generally, the ones that did not reach δ = 0 were set to δ = 0 by processing during the analysis. As shown in Figure 8a, the chemical shift at δ = 5.11 was the proton at the H1 position on BX; the chemical shifts in the range of δ = 3.3–4.5 were the proton peaks of H2–H5. Figure 8b shows that the double peaks with chemical shifts δ = 6.75 and δ = 6.77 were the proton peaks of H-2 and H-6, the chemical shift δ = 6.92 was the proton peak of H-4, the chemical shifts δ = 7.39 and δ = 7.40 were the proton peaks of H-2′ and H-6′, the chemical shifts δ = 6.80 were the chemical shifts of H-3′ and H-5′, and δ = 6.83 and δ = 6.95 were the proton peaks of Ha and Hb. As can be seen in Figure 8c, the new chemical shifts at δ = 0.85 were the proton peaks of CH_3_CH_2_— of EGDMA, and the chemical shift of the proton on —NH_2_ was δ = 2.63. As seen in Figure 8d, the chemical shifts of —OCOCH_3_ and —COCH_3_ appeared at δ = 0.99 and δ = 1.05, while the proton peaks of benzene rings appeared at δ = 7.96 to 8.41. Combined with the IR spectra, it was determined that EGDMA and 3-carboxyphenylboronic acid were successfully introduced into the product.

### 3.4. Molecular-Docking Study

A molecular-docking approach was used to simulate the structural unit of the product (3-CBA-BX/Res-g-EGDMA) with four receptor proteins screened from Protein Data Bank (http://www.rcsb.org/ accessed on 26 March 2022). The structures were optimized 100 times using a genetic algorithm, and the best docking parameters were selected for further analysis. In general, the lower the binding free energy is, the stronger the compound binds to the receptor, and the stronger the compound inhibits the receptor protein. From Table 1, it can be seen that the optimal binding free energies of 3-CBA-BX/Res-g-EGDMA to receptor proteins 2HE7, 5JQI, 5ZSY and 6YAU were −6.3, −3.24, −3.34 and −3.66 kJ/mol, respectively. The results indicated that 3-CBA-BX/Res-g-EGDMA and the four receptor proteins had high binding stability and a good docking effect, with the strongest binding ability with respect to 2HE7.

**Figure 9:**
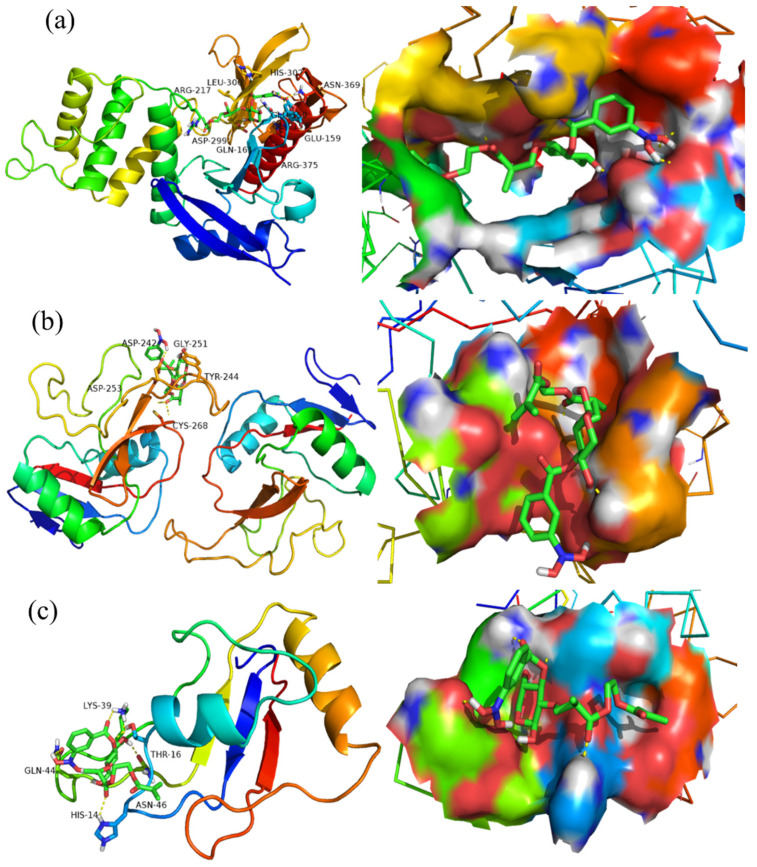

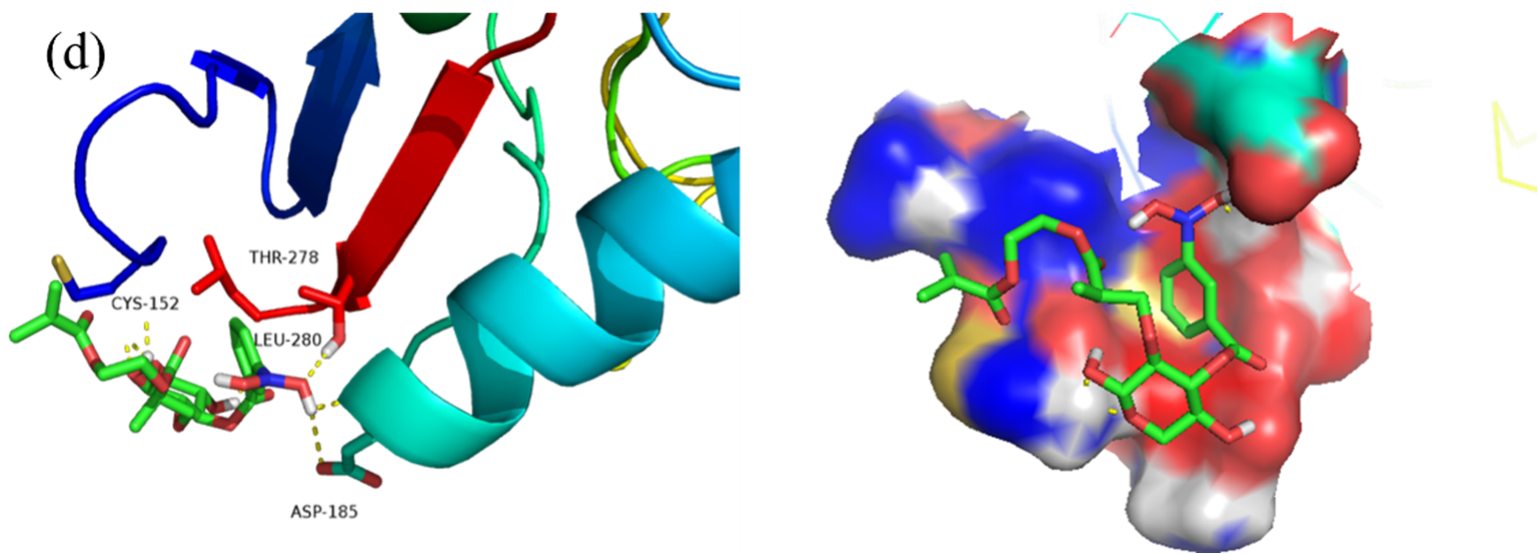
(**a**) The docking conformation figure of 3-CBA-BX/Res-g-EGDMA and 2HE7. (**b**) The docking conformation figure of 3-CBA-BX/Res-g-EGDMA and 5JQI. (**c**) The docking conformation figure of 3-CBA-BX/Res-g-EGDMA and 5ZSY. (**d**) The docking conformation figure of 3-CBA-BX/Res-g-EGDMA and 6YAU.

The best spatial conformation of 3-CBA-BX/Res-g-EGDMA binding to receptor proteins 2HE7, 5JQI, 5ZSY and 6YAU can be seen in Figure 9. Among them, receptor proteins 2HE7, 5JQI, 5ZSY and 6YAU are shown in cartoon form, while 3-CBA-BX/Res-g-EGDMA is shown in the ball-and-stick model, and the hydrogen bonds generated by the amino acid residues docked with the small molecule ligands are shown as yellow dashed lines. As can be seen from Figure 9, 3-CBA-BX/Res-g-EGDMA formed hydrogen bonds with amino acid residues HIS-302, LEU-300, ARG-217, ASN-369, GLU-159, ASP-299, GLN-161 and ARG-375 of 2HE7. It formed hydrogen bonds with amino acid residues ASP-242, GLY-251, TRY-244, ASP-253 and CYS-268 of 5JQI. It formed hydrogen bonds with amino acid residues LSY-39, GLN-44, THR-16, ASN-46 and HIS-14 of 5ZSY. It formed hydrogen bonds with amino acid residues CYS-152, THR-278, LEU-280 and ASP-185 of 6YAU.

The above analyses showed that 3-CBA-BX/Res-g-EGDMA and receptor proteins 2HE7, 5JQI, 5ZSY and 6YAU (where 2HE7 and 5ZSY are lung cancer proteins and 5JQI and 6YAU are liver cancer proteins) could form a strong hydrogen-bond network, which theoretically proved that 3-CBA-BX/Res-g-EGDMA had inhibitory and targeting effects on receptor proteins 2HE7, 5JQI, 5ZSY and 6YAU.

### 3.5. Inhibition Analyses of Tumor Cell

The inhibition rates of BX, BX/Res and 3-CBA-BX/Res-g-EGDMA at different mass concentrations were evaluated in cancer cells MGC80-3, NCI-H460, BEL-7407 and NGEC. The results (tested at Henan cancer hospital and the key laboratory of pharmaceutical chemistry and drug molecular engineering at Guangxi Normal University) are shown in Table 2.

The inhibition rates of BX, BX/Res and 3-CBA-BX/Res-g-EGDMA on human gastric cancer cells (MGC80-3), human lung cancer cells (NCI-H460), human liver cancer cells (BEL-7407) and normal gastric mucosa epithelial cells (NGEC) were evaluated. The results of the inhibition rates showed that BX and BX/Res showed weak inhibition of the above three cancer cells at concentrations equal to or higher than 50 μg/mL; however, 3-CBA-BX/Res-g-EGDMA showed strong inhibition at low concentrations (1 μg/mL). Moreover, the oncogenic effect significantly increased with the loading of more products. 3-CBA-BX/Res-g-EGDMA at 100 μg/mL could inhibit MGC80-3 cancer cells up to 36.71 ± 4.93%, which was about 18 times higher than BX. Although the inhibition rate of 3-CBA-BX/Res-g-EGDMA on NCI-H460 and MDA-MB-231 was not as strong as that of MGC80-3, it still had a strong inhibitory effect; in addition, the growth inhibition rate of NCI-H460 cancer cells was 25.87 ± 3.28%, which was about 6 times that of BX, and the growth inhibition rate of MDA-MB-231 cancer cells was 20.76 ± 2.13%, which was about 20 times that of BX. In addition, for normal cells, 3-CBA-BX/Res-g-EGDMA showed low toxicity.

## 4. Conclusions

A BX/Res-g-EGDMA copolymer was synthesized via graft copolymerization reaction between BX and Res under optimized process conditions, and *G* could reach 142.44%. On this basis, a 3-CBA-BX/Res-g-EGDMA derivative was obtained via esterification reaction, with 3-CBA as the esterifying agent, and *DS* was 0.485. The products were structurally characterized using FTIR, XRD, TG–DTG, SEM and 1H NMR. The results showed that the 3-CBA-BX/Res-g-EGDMA had a spherical structure with an average particle size of about 100 nm, and its crystalline structure and thermal stability were different from those of the raw materials.

The antitumor activity of 3-CBA-BX/Res-g-EGDMA nanoparticles was examined by docking the optimal molecular conformation to the receptor protein with the molecular-docking technique. The results showed that 3-CBA-BX/Res-g-EGDMA showed good docking activity against four tumor cell proteins with strong binding energy and docking sites. Furthermore, the MTT assay clearly demonstrated that the complex-modified derivatives had strong inhibitory effects on gastric cancer cells, hepatocellular carcinoma cells and lung cancer cells. The novel 3-CBA-BX/Res-g-EGDMA showed an inhibition rate of 36.71 ± 4.93% against gastric cancer cells, while it was almost non-toxic to normal cells.

Based on these results, it is hypothesized that 3-CBA-BX/Res-g-EGDMA nanoparticles have good antitumor activity. This research project can stimulate more scholars to explore and more deeply study theoretical and practical aspects. It provides new research directions for the development of natural, green and safe antitumor drugs, drug carriers and functional materials.

## Figures and Tables

**Table 1:** Binding free energy of BX and 3-CBA-BX/Res-g-EGDMA.

Sample	PDB Code			
	2HE7	5JQI	5ZSY	6YAU
ΔE (BX)	−4.65	−3.82	−3.92	−4.44
ΔE (3-CBA-BX/Res-g-EGDMA)	−6.3	−3.24	−3.34	−3.66

**Table 2:** The inhibition ratio of BX, BX/Res and 3-CBA-BX/Res-g-EGDMA on different cancer cells and normal cells.

Sample	Mass Concentration (μg/mL)	Inhibition Ratio (%)			
		NGEC	MGC80-3	BEL-7407	NCI-H460
BX	100	1.81 ± 0.47	2.02 ± 0.57	1.07 ± 0.71	4.62 ± 2.79
	50	1.64 ± 0.69	0.24 ± 0.08	1.18 ± 0.34	0.71 ± 0.22
	20	−0.31 ± 0.52	−0.15 ± 0.13	0.35 ± 0.26	−0.24 ± 0.19
	10	−3.07 ± 0.33	−2.99 ± 1.11	0.47 ± 0.29	−2.97 ± 1.43
	1	−5.78 ± 0.20	−3.27 ± 1.61	−0.45 ± 0.31	−4.33 ± 2.03
BX/Res	100	1.53 ± 0.42	3.43 ± 1.35	2.39 ± 0.58	6.81 ± 2.03
	50	1.31 ± 0.57	2.14 ± 0.19	2.25 ± 0.51	4.37 ± 2.64
	20	−0.58 ± 0.33	1.57 ± 0.52	1.62 ± 0.65	2.86 ± 0.41
	10	−3.45 ± 0.41	0.76 ± 0.26	0.87 ± 0.26	1.25 ± 0.36
	1	−5.80 ± 0.54	−1.34 ± 0.27	0.14 ± 0.07	0.62 ± 0.37
3-CBA-BX/Res-g-EGDMA	100	2.91 ± 0.57	36.71 ± 4.93	20.76 ± 2.13	25.87 ± 3.28
	50	1.71 ± 0.84	32.38 ± 4.16	16.39 ± 3.72	21.93 ± 4.41
	20	1.37 ± 0.62	28.84 ± 3.79	11.87 ± 3.21	17.38 ± 3.65
	10	−0.98 ± 0.26	21.61 ± 2.83	6.95 ± 2.28	11.65 ± 3.01
	1	−1.86 ± 0.59	16.15 ± 3.61	4.63 ± 1.37	7.37 ± 2.36

## Data Availability

Not applicable.
